# Photoablative cosmetic iridoplasty: effective, safe, and predictable—eye color change in 1176 eyes

**DOI:** 10.1007/s10792-021-01693-5

**Published:** 2021-01-23

**Authors:** Pedro Grimaldos Ruiz

**Affiliations:** Eyecos Clinic, Tuset, 23-25, 08006 Barcelona, Spain

**Keywords:** Photoablative cosmetic iridoplasty, Eye color change, Iris heterochromia, Iris nevus, Ocular cosmetics

## Abstract

**Purpose:**

To evaluate photoablative cosmetic iridoplasty (PCI), and its efficacy, safety, predictability, and satisfaction with the 532 nm Crystal Q-switched Nd: Yag laser, with 3–4 ns pulses, for depigmentation of the anterior epithelium of the iris in cases of heterochromia, nevus, or cosmetic indications (eye color change).

**Design:**

Prospective clinical study on efficacy, safety, predictability, and satisfaction.

**Method:**

The selection of patients was carried out in healthy individuals, over 18 years of age, with iris heterochromia (congenital-7% or acquired, secondary to topical medication-1%, trauma-0.5% or surgery-0.25%), nevus-0.25% and cosmetic cases-91%. Data were collected independently by assistant optometrists and classified in database. Excel statistical program was used to perform a general descriptive study, calculation of correlation factors, and statistical significance analysis between quantitative variables (Student *T* Test). PCI was performed in 1176 eyes of 588 patients. The procedures were planned in 2–3 phases of 4 consecutive sessions spaced 4–6 months apart. The IRÎZ^®^ (Eyecos^®^) scanner was used to evaluate the cases, with photography, optical coherence tomography, and pneumotonography modules, along with the following software programs: Predictor^®^, Simulator^®^ 3D, Analyzer^®^ and Planner^®^ (Eyecos^®^).

**Results:**

This study began in 2012, so far 9 years of follow-up, to compare and choose the most suitable among 4 types of lasers to perform cosmetic iridoplasty. Finally, after 5 years, the Crystal Q-switched Nd: Yag at double frequency (532 nm) with 3–4 ns pulses demonstrated the highest efficacy, safety and predictability, so since early 2017 only this equipment has been used. Significant differences were found after 5-year follow-up between 1064, 532, 577 and 532/3–4 ns *p* = 0.09172, 0.06377 and 0.10183. From 9 January 2017 to 28 February 2020, 1176 eyes have been treated in 588 patients, with a mean age of 33.7 years (SD = 9.68 years, range = 18–70 years). 46.2% were male, and 53.7% were female. The efficacy, as quantified with the Analyzer^®^ comparison software, was nearly 87–95%. There were no significant differences in corrected vision (9 years total follow-up *p* = 0.78235; last 4 years FU *p* = 0.99999) and ocular pressure (9 years total FU *p* = 0.68251; last 4 years FU *p* = 0.63204) before and after the procedure. The only notable complications (25%) were delayed and brief iritis, which were self-limited with routine topical treatment. The predictability was 80–90%. In the lightest-colored eyes, turquoise blue colors were obtained as a rule, in varying brightness; and in the darkest ones, gray blue tones of varying lightness. The patients’ subjective satisfaction at the end of treatment was 95%.

**Conclusion:**

After 9 years of uninterrupted follow-up, PCI has demonstrated a high effectiveness to selectively depigment superficial melanin of iris, with a high predictability and patient satisfaction, without remarkable long-term complications. Only for a week, appropriate pre- and postoperative medication was necessary to guarantee the absence of discomfort, thus confirming security. PCI is effective, safe, and predictable for the treatment of pigmentary disorders in the iris and for the elective cosmetic indications in eye color change.

## Introduction

Pigmentary disorders of the iris (heterochromia) can be congenital or secondary, due to medical iatrogenesis, metabolic diseases, trauma, or complications from ocular surgeries. Until now, they have been treated conservatively with contact lenses and through surgeries, with cosmetic intraocular lenses or keratopigmentation. However, these invasive techniques have caused frequent and serious complications such as glaucoma, uveitis, endothelial or corneal damage, reduction in vision or the visual field, aberrations, etc. Furthermore, the aesthetic effect obtained is always artificial (“robot eyes”), with fixed pupils that do not react to light, synthetic colors and parasitic reflections [1–13].

Therefore, Ophthalmology lacked an effective and safe technique that was capable of treating pigmentary disorders of iris, as well as purely cosmetic indications. The new procedure had to be reliable in the long term and achieve patient satisfaction. After several years of basic research on intraocular lenses and intracorneal implants, we finally dismissed their viability. For this reason, we planned the development of the cosmetic iridoplasty by laser, the first technique in history able to guarantee all the needed requirements.

In 2011 we began a research and development project on a laser iris depigmentation technique. Lasers’ depigmentation potential when used in the anterior segment had been known for decades [14–20], so we decided to perform a comparative study of four types of equipment: Crystal Q-switched Nd: Yag (1.064 nm), Crystal Q-switched Nd: Yag at double frequency (532 nm), semiconductor optical pump laser (577 nm) and the Crystal Q-switched Nd: Yag at double frequency (532 nm) with 3–4 ns pulses. These lasers are routinely used to perform iridotomy, capsulotomy, synechiotomy, suture lysis, membranotomy, iridoplasty, gonioplasty, pupilloplasty, and trabeculoplasty, in traumatic pathologies, congenital malformations, glaucoma, and following glaucoma and cataract surgeries [14–16].

In 2012, we performed a histological study in 100 cadaver eyes to familiarize ourselves with the characteristics of the melanin in the iris and its distribution in different grades of ocular pigmentation, as well as to test the effects of the four types of laser on the iris. A pilot clinical study was also performed in three patients, with one year of monitoring, to test the efficacy and safety of the procedure in humans.

Between 2013 and 2016, once the absence of serious or irreversible secondary effects was confirmed, 1.328 eyes were studied in patients without general or ocular diseases, and without psychiatric or personality disorders. The treatments were planned sequentially, in two or three phases separated by at least four months, which allowed the monitoring of patients and the evaluation of results and complications.

The four lasers demonstrated good midterm and long-term safety (> 90%) without any reported cases of vision loss. Best corrected visual acuity (*p* = 0.78235) and intraocular pressure (*p* = 0.68251) showed no significant differences after the treatments. However, upon comparing the four lasers we found short-term and long-term differences in efficacy, safety, and predictability. After 5-year follow-up, the global statistical difference between the 1064 and 532 nm/3–4 ns laser was *p* = 0.09172; between 532 and 532 nm/3–4 ns it was *p* = 0.06377; and between 577 and 532 nm / 3-4 ns it was *p* = 0.10183.

**Fig. 1:**
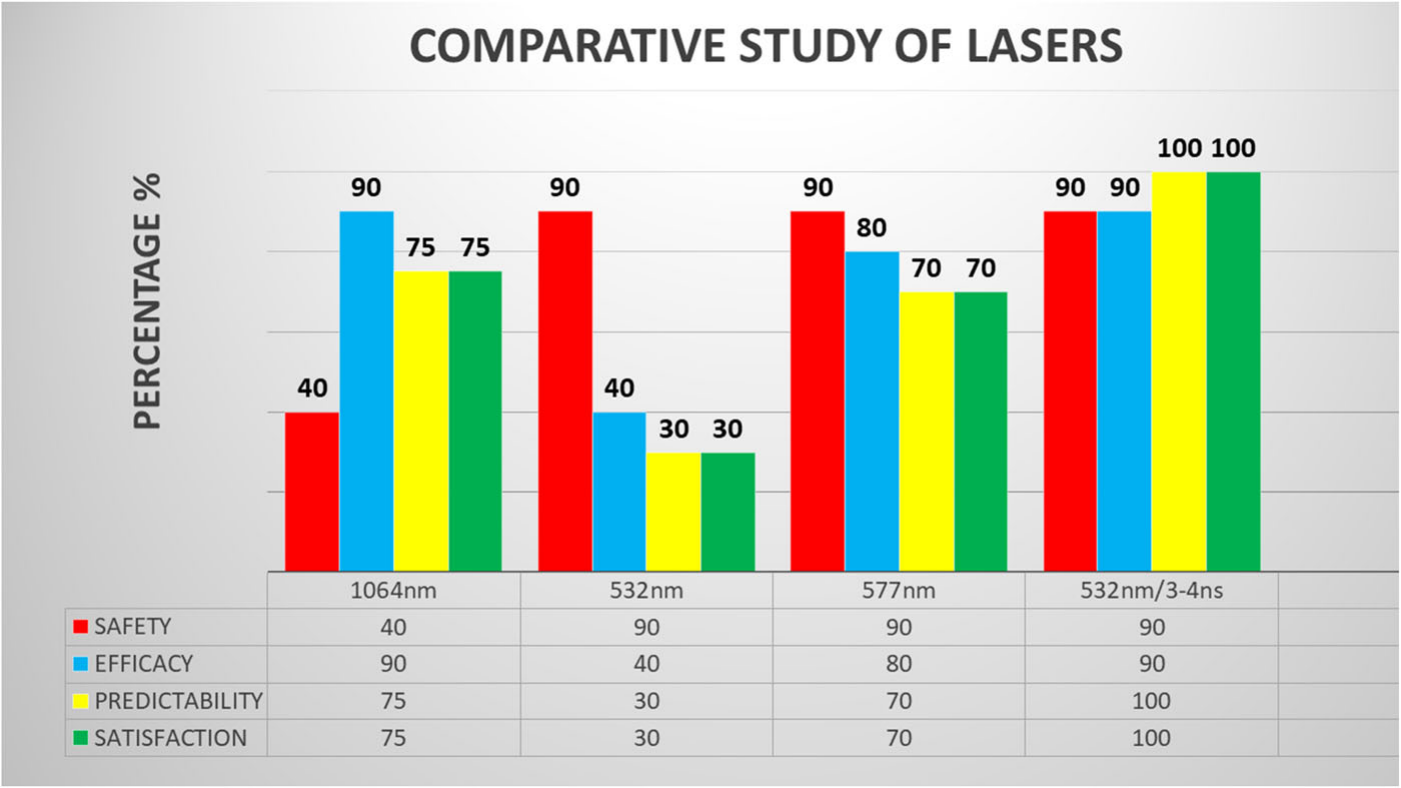
Comparison of safety, efficacy, predictability, and satisfaction, among Nd: Yag (1064 nm), Nd: Yag (532 nm), Optical Pump Semiconductor (577 nm) and Crystal Q-switched Nd: Yag (532 nm/3–4 ns) lasers. Scale from 0 to 100 percentage (%). The 532-nm Crystal Q-switched Nd: Yag laser with 3–4 ns pulses showed the best levels in efficacy, which was almost immediate (90%), safety (90%), with minimal secondary effects, and short-, mid-, and long-term high predictability: 90–95.5%. After 5-year follow-up, the global statistical difference between the 1064 nm and 532 nm/3–4 ns laser was *p* = 0.09172; between 532 and 532 nm/3–4 ns it was *p* = 0.06377; and between 577 and 532 nm/3–4 ns it was *p* = 0.10183

The 1.064-nm Nd: Yag laser with a photo-disruptive mechanism showed a very high immediate efficacy (90%), and spectacular cosmetic results. However, the safety at 24 h was fairly low (40%), as it produced pressure spikes, blurry vision, and frequent discomfort. Furthermore, the long-term predictability was imprecise due to delayed scarring phenomena (75%). The initial efficacy of the two lasers with a photo-thermal mechanism, 532 nm and 577 nm, was low and enough (40% and 80% respectively), and although the safety was very high (> 90%), they lacked predictability (30% and 70% respectively). The 532-nm Crystal Q-switched Nd: Yag laser with 3–4 ns pulses showed the best levels in efficacy, which was almost immediate (90%), safety (90%), with minimal secondary effects, and short-, mid-, and long-term high predictability: 90–95.5% (Fig. 1). Therefore, beginning in 2017, we decided to only use this laser for all iris depigmentation treatments. Furthermore, in 2017, Yildirim et al. published: “Evaluation of color-changing effect and complications after Nd: YAG laser application on iris surface”. An experimental study in rabbits, that confirmed good cosmetic results and safety [21], and also, in 2018, 1 year later, Basoglu et al. [22] published: “The effect of SLT laser application on iris to treat sectorial heterochromia: A promising technique”.

The objective of our study was finally focused to evaluate the efficacy, safety, and predictability of the 532-nm Crystal Q-switched Nd: Yag laser, with 3–4 ns pulses, for iris depigmentation treatment in cases of congenital and acquired heterochromia, nevus and for elective cosmetic indications in eye color change.

## Methods

The selection of patients was carried out in healthy individuals, over 18 years of age, with iris heterochromia (congenital-7% or acquired, secondary to topical medication-1%, trauma-0.5% or surgery-0.25%), nevus-0.25% and cosmetic cases-91%.

The exclusion criteria were: under 18 years old, personal or family history of glaucoma, chronic ocular pathology (uveitis, iritis, retinopathy, trauma), systemic inflammatory, infectious, or oncological diseases, chronic vascular diseases (diabetes, Raynaud), autoimmune diseases (rheumatoid arthritis, Crohn’s disease, ulcerous colitis, Behçet disease, lupus erythematosus, multiple sclerosis), serious psychological disorders or psychiatric diseases (depression, bipolar disorder, obsessive, compulsive, or paranoid patients or, in particular, those with body dysmorphia disorder). Specific cases of medical allergies or intolerances were also rejected, as well as the chronic consumption of anabolic, steroids, hormones, or drugs.

**Fig. 2:**
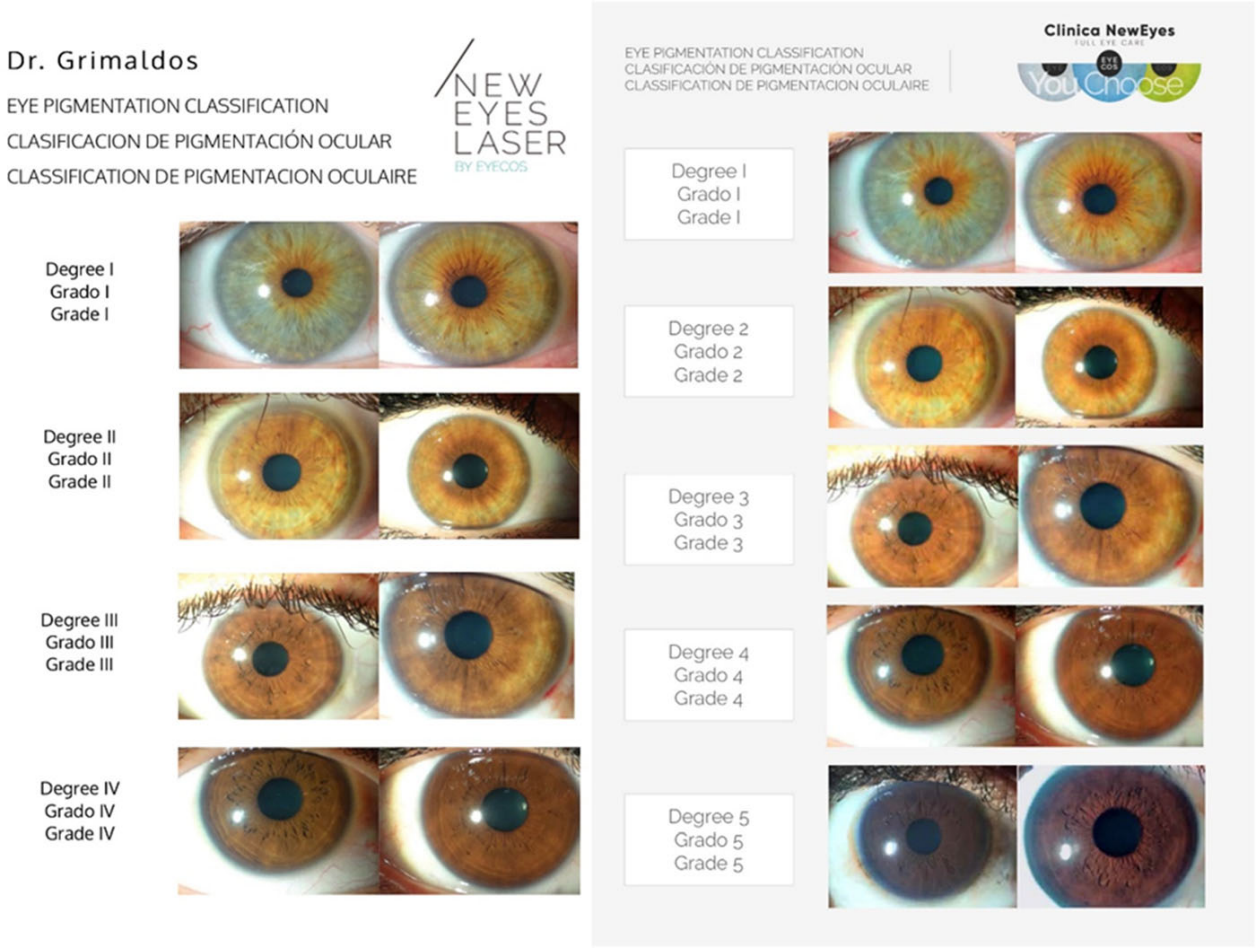
Levels of ocular pigmentation by Eyecos. On the left, first classification with four grades. On the right, current classification with five grades of pigmentation. Since 2017, with the exclusive use of the Nd: Yag 532 nm (3–4 ns) laser, grades IV of pigmentation were admitted as candidates, so a second classification was necessary, incorporating very dark eyes (grades V), to establish the new admission limit

Regarding degrees of pigmentation, prior to 2019 only cases in grades I–III of the Eyecos ocular pigmentation classification (Fig. 2) were admitted, but in the final months and at present we began to accept grade IV cases, with very satisfactory results.

The indications to apply PCI have been alterations in iris pigmentation, which have included unilateral or bilateral, partial or complete, congenital heterochromia**,** single or multiple nevus, and acquired causes, secondary to trauma, surgical complications (cataracts), and due to iatrogenesis from the abuse of prostaglandin eye drops to lengthen eyelashes. The differential diagnosis between nevus and iris melanoma was performed using three methods: 1—color assessed with the IRÎZ Scanner (clear in nevus and almost black in melanomas); 2—thickness and stromal infiltration measured by OCT (nevus are superficial and do not infiltrate the stroma; but melanomas are very thick and deeply infiltrate the stromal layer); and 3—if still in doubt, an iris angiography can be performed (in nevus the pattern is like normal tissue; but in melanomas, a large nutrient vessel with strong extra vascular diffusion of fluorescein is easily seen). In relation to the patients who used prostaglandin drops, all of them were young and healthy. None of them had previous glaucoma, but the reason was only cosmetic for the lengthening of the eyelashes (Latisse^®^). Therefore, the choice of these candidates did not alter the validation of their results.

The most frequent reasons for interested patients were purely aesthetic (close to 91% of the total), without the presence of heterochromia. It surprised us that the stated reasons were not capricious, but rather a professional need (contract requirements for image professionals like models or actors), sentimental (specific preferences of their partner) and psychosocial. The latter group was without a doubt the largest, consisting of individuals who had spent their whole life wanting the same color eyes as their family members (parents and siblings with blue or green eyes while theirs are brown), or people subject to a social discrimination based on race, who saw this technique as the solution to the issue. In 95% of the cases, the patients came after many years of seeking information from the clinic, over the phone or via email, in patient forums, social networks, and through direct contact with the doctor. Therefore, they had comprehensive information, including a detailed informed consent, and the analyses in pre- and post-laser treatments. None of the cases were compulsive or poorly considered decisions.

### Photoablative cosmetic iridoplasty (PCI) procedure

**Fig. 3:**
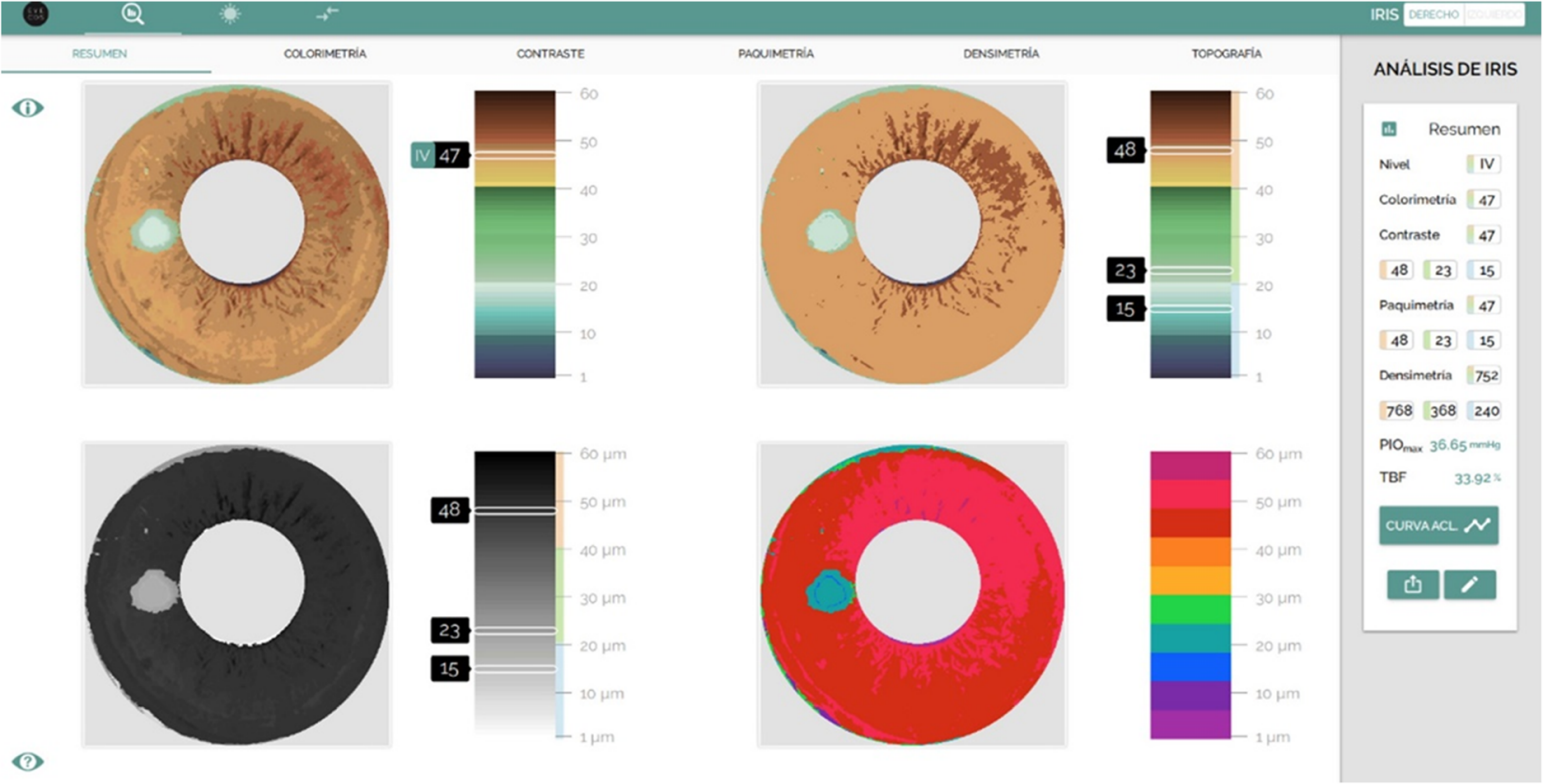
Eyecos iris summary: pigmentation grade, colorimetry, color contrast, pachymetry, topography and physio-dynamic parameters of aqueous humor (maximum pressure, clearance curve and trabecular blocking factor). Using Eyecos IRÎZ Scanner with the Analyzer program, we automatically obtain 4 graphs that describe the anterior pigmentary layer of the iris. In addition, Analyzer calculates the essential physio-dynamic parameters of aqueous humor in the anterior chamber, to guarantee the safety of the procedure. The Eyecos Iris Summary is the key piece to perform accurate PCI procedures. The example shows a typical case of grade 4 pigmentation with a value of 47 out of 60 (top left). The contrast calculates the average of the brown tones which is 48, 23 for green and 15 for blue (top right). In the lower left, we see the pachymetry data, and on the lower right the topography data. In the 4 diagrams a whitish spot appears corresponding to the reflection of the flash, which has been eliminated in the latest version of the Analyzer program. In the data sidebar we can see the densitometry values: 768 for brown, 368 for green, and 240 for blue (expressed in mg/100 ml on a scale of 0–960 mg/100 ml). In the lowest part, we see the maximum ocular pressure value that would be reached with the complete removal of the iris surface (36.65 mmHg). Below the value of the Trabecular Blocking Factor (FBT = 33.92%). Finally, we can access to the Clearance Curve

A detailed clinical history was gathered, especially focused on detecting exclusion criteria. If they were determined to be a good candidate, a general in-depth ophthalmologic examination was performed, as well as another specific one to PCI. We used the IRÎZ^®^ (Eyecos) scanner and Analyzer program, which provided us with the data required to adequately plan the treatment: pigmentation grade, colorimetry, color contrast, iris pachymetry, and 3D topography. The scanner consists of three modules: photography (Topcon SL-D), optical coherence tomography (OCT Topcon SL-SCAN1) and pneumotonography (Reichert M30). The Analyzer IRÎZ (Eyecos) software also calculates the physio-dynamic parameters of the aqueous humor in the anterior chamber, which are indispensable for a safe technique: maximum intraocular pressure (IOP max), clearance curve (CC) and trabecular blockage factor (TBF). These data are shown in a simplified format in the Eyecos Iris Summary (Fig. 3).

After obtaining the Iris Summary (Eyecos), we used the Predictor IRÎZ software (Eyecos) to show the patient the expected results and their probability, based on the pigmentation of their eyes, skin, and hair, and that of their parents. This program is based on phenotype calculations according to the two genes law of eye color, in which a B allele (Brown) is dominant over a G allele (Green), which is in turn dominant over b (blue). By combining the variables, Predictor gives us the most probable option, as a percentage, followed by the second alternative. To achieve a realistic effect, the program offers real examples of eyes with similar characteristics that have been treated before. If the patients don’t know their parents (adoption or premature death), it is possible to perform a genetic test (Eyecos Color Biochip) using a saliva swab, which within a few weeks will give us a detailed report on their eye color genes, and the prediction of the results. The genetic test for open angle glaucoma is also available.

Afterward, we used the Simulator 3D IRÎZ software (Eyecos) to perform a 3D simulation of the patient’s eye and their most probable results. Furthermore, this program can simulate the effect that various lighting levels will have on the diameter of the pupil, as well as the influence of observation distance on the final color effect obtained. At this point, if the patient is considered eligible and has all the necessary information, they are required to sign the informed consent for PCI. After importing the Eyecos Iris Summary data from the Analyzer software, the Planner IRÎZ software (Eyecos) schedules each laser session and the different phases needed through treatment completion.

The medication prior to the procedure includes dexamethasone, tobramycin, and timolol maleate drops at 0.50% every 8 h starting two days before. As we have known for many years, the application of laser to the iris causes an inflammatory reaction similar to iritis, and laser depigmentation of the trabeculum in the SLT technique generates pressure peaks after its performance. To prevent both acute complications, previous therapy with topical anti-inflammatory and antihypertensive drugs is indicated. Tobramycin drops are associated just to prevent the appearance of conjunctival infection secondary to the repeated instillation of eye drops and excessive manipulation of the eyes during the 4 consecutive days that the PCI lasts, which would make it difficult to complete.

Without the routine administration of oral anxiolytics, anesthetic drops of tetracaine and oxybuprocaine are administered. If necessary, pilocarpine 2% drops can be applied to keep pupils miotic during the procedure. VOLK capsulotomy or iridectomy lenses are used, with methylcellulose drops, to fix the ocular globe and optimize focus in the iris.

All treatments were performed with the 532-nm Crystal Q-switched laser, with 3–4 ns pulses (commonly used in selective laser trabeculoplasty, or SLT). Models from different manufacturers were used (Ellex, Lightmed, Quantel Medical, Lumenis). The Planner software provides the parameters we must use according to the Analyzer software’s Iris Summary: power, duration, repetition frequency, number of shots, and treatment area. We follow the guidelines indicated by Planner until the specific area of the iris has been treated. The sessions tend to take no more than 5 min. The laser's effects are painless, although the light from the slit-lamp causes discomfort. Each phase consists of four or five consecutive daily sessions, which are repeated every 4–6 months until finalization, with the treatment completed in two or three phases.

Following the procedure, both steroidal and non-steroidal oral anti-inflammatories are prescribed, and the frequency of eye drops is increased to every hour for the first 5 h, and then every 2 h until midnight. Following the final session, the dose decreases to three times a day for only 1 week, and then we add artificial tears with sodium heparin every 8 h for 3 months. The topical anti-inflammatory and antihypertensive treatment lasts only one week, to avoid any type of discomfort. Its use does not affect the safety assessment of PCI since the statistical significance study is performed between the patient's previous values and those found in the medium and long term, when the patient no longer uses any topical treatment. Follow-up of visual acuity, refraction error, Goldmann eye pressure, biomicroscopy, HQ photography, and colorimetry study are carried out weekly, monthly and then every 4 months.

The patient is required to submit photographs of their eyes every 1–2 months using the Eye Selfie^®^ (Eyecos) application to monitor their progress. Additionally, the patients receive an ocular examination in each phase, and the IRÎZ Scanner is used in order to calculate new Iris Summaries and allow comparison with previous progress. One function lets us automatically calculate the differences obtained in grade, colorimetry, contrast, pachymetry, topography and physio-dynamic parameters of the aqueous humor. In this way, patients are certified of the efficacy, safety, and predictability of the PCI through a written or digital report.

Data were collected independently by assistant optometrists and classified in database. Excel statistical program was used to perform a general descriptive study, calculation of correlation factors, and statistical significance analysis between quantitative variables (Student *T* Test).

## Results

From 9 January 2017 to 28 February 2020, 1176 eyes have been treated in 588 patients, with a mean age of 33.7 years (SD = 9.68 years, range = 18–70 years). 46.2% were male, and 53.7% were female. The initial refractive errors were: emmetropia (43.25%), myopia, with or without astigmatism (43.75%), and hypermetropia, with or without astigmatism (13%). 89% presented visual acuity with correction of 0.9–1.0. The mean Goldman tonometer ocular pressure was 10.67 mmHg (SD = 1.06 mmHg). Concerning patient history, 10.5% of the patients had received refractive surgery (the majority received LASIK, and a minority received PRK), 2% had posterior chamber intraocular lenses due to cataract surgeries, 2% presented different types of heterochromia, 1% had corneal leukomas, and 8.5% had existing systemic pathologies that were not considered exclusion factors.

**Fig. 4:**
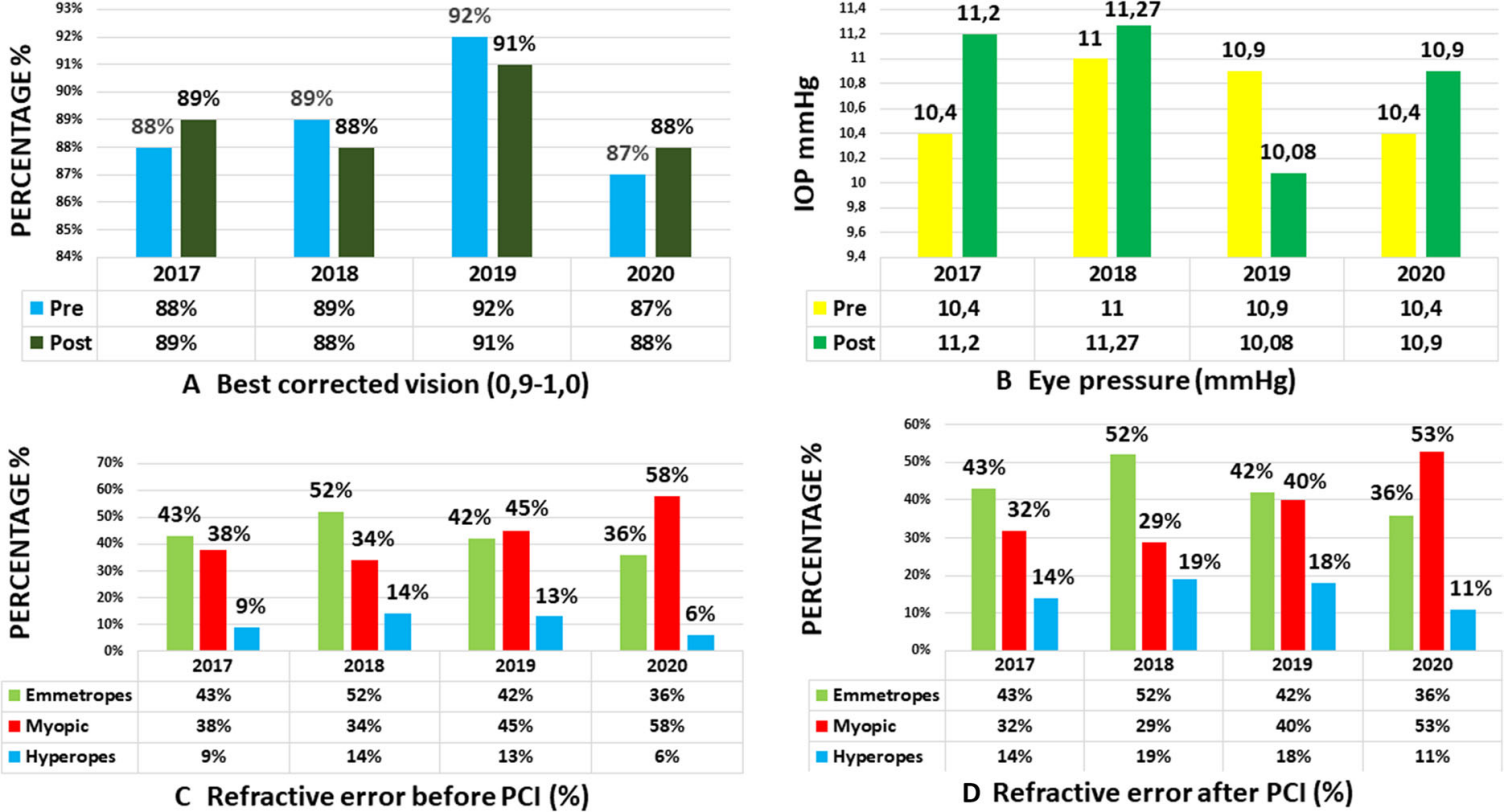
**a** Best corrected vision (0.9–1.0), before and after PCI. No significant differences were found (9 years total follow-up *p* = 0.78235; last 4-year follow-up *p* = 0.99999). **b** Eye pressure before and after PCI. No significant differences were found (9 years total follow-up *p* = 0.68251; last 4-year follow-up *p* = 0.63204). **c** Percentage of emmetropes, myopic, and hyperopes, before PCI. **d** Percentage of emmetropes, myopic, and hyperopes, after PCI. Hyperopic shift. No significant differences were found on emmetropes after 9 years total follow-up *p* = 0.34659; and last 4-year follow-up *p* = 0.99999. Significant differences were found on myopia after 9 years total FU *p* = 0.02356; and last 4 years FU *p* = 0.00023. Significant differences were found on hyperopia after 9 years total FU *p* = 0.00054; but not after last 4 years FU *p* = 0.99999)

The series studied was highly safe, since it is planned as a sequential procedure in various phases, spaced out over 4–6 months, with adequate medication before and after each session. Prior to the treatment, visual acuity of 0.9–1.0 with best correction was 89%, which did not change after the treatment (*p* = 0.99999) (Fig. 4). The best corrected vision has shown slight differences in the annual controls, but this has been due to hyperopic shift secondary to the laser and the failure to update glasses and contact lenses, due to small differences in prescription. The mean ocular pressure prior to PCI, using a Goldman tonometer, was 10.6 mmHg, and was 10.8 mmHg following the procedure (*p* = 0.63204) (Fig. 4). We have found that the long-term trend of eye pressure without any type of treatment has been slightly downward, never upward. We believe it is due to the increase in intraocular volume and chamber angle after removal of the superficial layer of melanin from the iris.

The refractive defects prior to PCI were: 43.25% emmetropia, 43.75% myopia, 13% hypermetropia. Following PCI, the percentages were 43.70% emmetropia, 38.5% myopia and 17% hypermetropia (Fig. 4). After 4-year follow-up, no significant differences were found on emmetropia *p* = 0.99999; significant differences were found on myopia *p* = 0.00023; but not significant differences were found on hyperopia *p* = 0.99999.

The refractive errors show a slight hyperopic shift, of + 0.50/+ 0.75 diopters. This phenomenon implied a slight decrease in the number of myopic patients, with or without astigmatism, no change in the percentage of emmetropic patients, and a small increase in hyperopic patients. Some myopic patients became emmetropic and, proportionally, several emmetropes became slightly hyperopic and presbyopic. For practical purposes, this change in refractive errors meant many patients with slight accommodative myopia no longer needed glasses or contact lenses, since their distance vision was ostensibly improved. But, on the contrary, hypermetropia and presbyopia increased in those over 40–45 years old, who began to need reading glasses or an increased prescription.

The 532-nm Crystal Q-switched Nd: Yag laser (3–4 ns) has a mixed photo-disruptive effect (like the Nd: Yag 1064-nm) and photo-thermic effect (532 nm/577 nm), and therefore generates the typical complications of both types of equipment, but much more limited, due to the large spot size (400 µm versus 8 µm in the Yag 1064-nm), and its short discharge time (3–4 ns, versus the milliseconds of solely thermal lasers). With adequate medication and distribution in phases, peaks in pressure are not observed following the sessions, nor decreased vision or tissue debris in the anterior chamber, and discomfort and pain are minimal.

The most commonly observed secondary effect, though it is brief and self-limiting, is the appearance of reactive iritis (25% of patients), which is often a week delayed. The symptoms are typical of acute iritis, with painful photophobia and miosis, which abates within 24–48 h with steroidal and mydriatic eye drops. On rare occasions, we can see transitory alterations in the morphology of the pupil, such as anisocoria or mydriasis, which spontaneously resolve within a few weeks, or with the help of B-complex vitamins.

Chronologically, the complications that appear in PCI are mostly acute and do not last over time. During the 4 consecutive days of treatment, patients may experience slight pain during the application of the laser and up to 2 h afterward. Blurred vision lasts only 2 h. The sensitivity to light and redness is variable according to each case, and increases a little on the fourth day. After finishing the laser sessions, an iritis reaction with photophobia, pain and redness of the eye may appear, but easily controlled with topical treatment. Vision may be a little blurry for several weeks due to scarring of the iris sphincter and dilator muscle, but it never lasts longer than 3–4 weeks. Pupillary deformities are self-limited. Later, after 4 weeks, no complications are reported.

An assessment of the efficacy and predictability was possible thanks to the use of the IRÎZ scanner and Iris Summary. The comparative evolution study function provides an exact quantitative analysis of the pigmentation grade, colorimetry, contrast level, pachymetry, and topography.

**Fig. 5:**
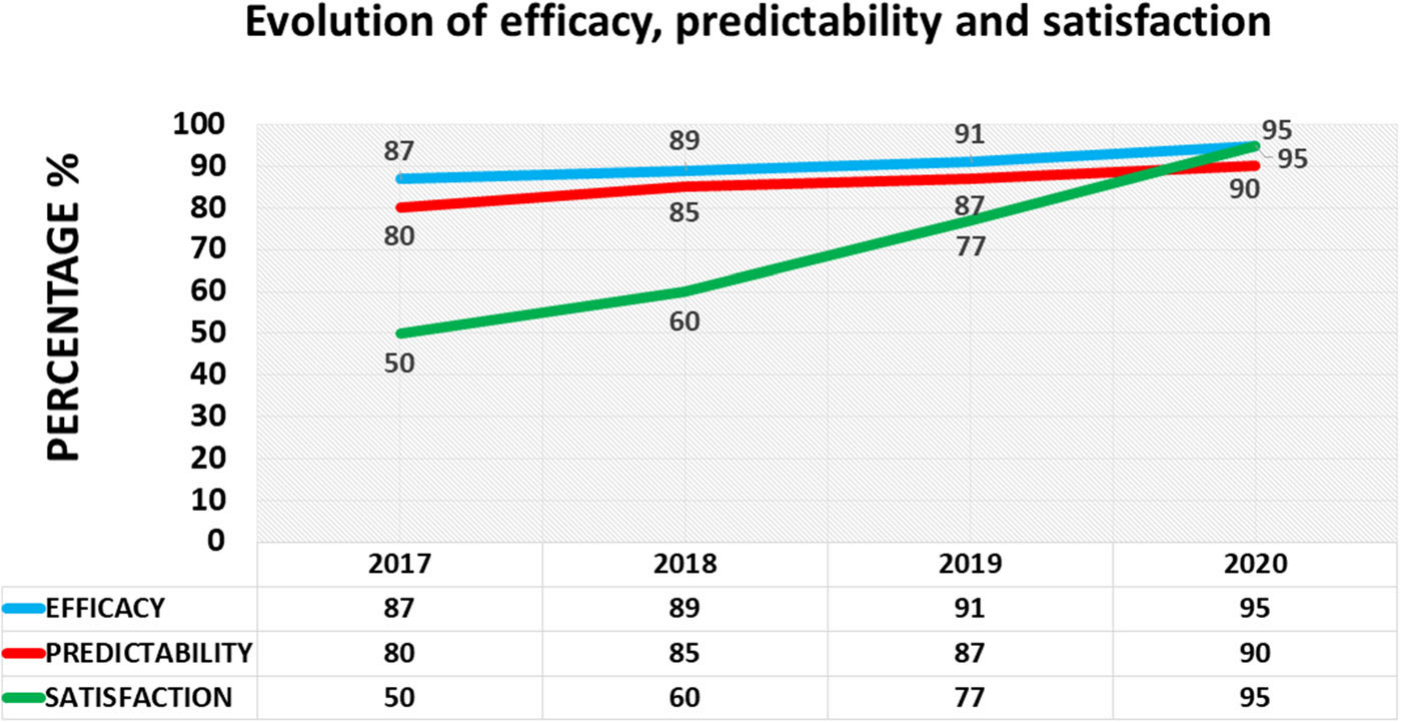
Evolution (last 4-year follow-up) of the efficacy, predictability, and satisfaction of PCI (%) with Nd: Yag 532 nm laser (3–4 ns). At the beginning, the efficacy and predictability are very high, although satisfaction is lower, but progressively rising to very high levels too (95%), at the end of the treatment. Significant difference was found between efficacy and satisfaction *p* = 0.09247; and lower between predictability and satisfaction *p* = 0.15403

The laser’s photoablative effect is immediately visible in grades I, II, and III, but not in the darkest colors, grade IV. For that reason, the efficacy is very high from the first phases (87%) to the last (95%) (Fig. 5). To calculate the precise predictability, we also use the comparison function of the Analyzer software (Eyecos), calculating the differences in colorimetric parameters, between the predicted result according to the Predictor software (Eyecos), which is based on the eyes of previously treated patients, and the real results obtained at the end of treatment. The predictability findings were very high from the beginning (> 90%), and at present the prediction is very simple and reliable (Fig. 5).

Paradoxically, the patients’ subjective satisfaction level does not follow the same pattern as the efficacy and predictability, even though the final result was well predicted at the beginning. Since the treatment is performed in phases, the brightened area is small in the beginning, and other areas have brown or yellowish melanin in the iris. Therefore, the light that hits the eyes mostly reflects the tones of pigmentation, but not the blue ones. The subjective satisfaction level doesn’t depend so much on the patient’s own opinion, but rather on comments from their family or friends, and the effect is not maximized until the treatment is complete. Therefore, the satisfaction curve is ascending, low in the beginning and very high at the end (95%). Significant difference was found between efficacy and satisfaction *p* = 0.09247; but lower between predictability and satisfaction *p* = 0.15403 (Fig. 5). Fortunately, the latest laser version has allowed us to accelerate the aesthetic effect, and then patient satisfaction curve reaches very high levels just after the first laser phase, in 3 or 4 weeks.

**Fig. 6:**
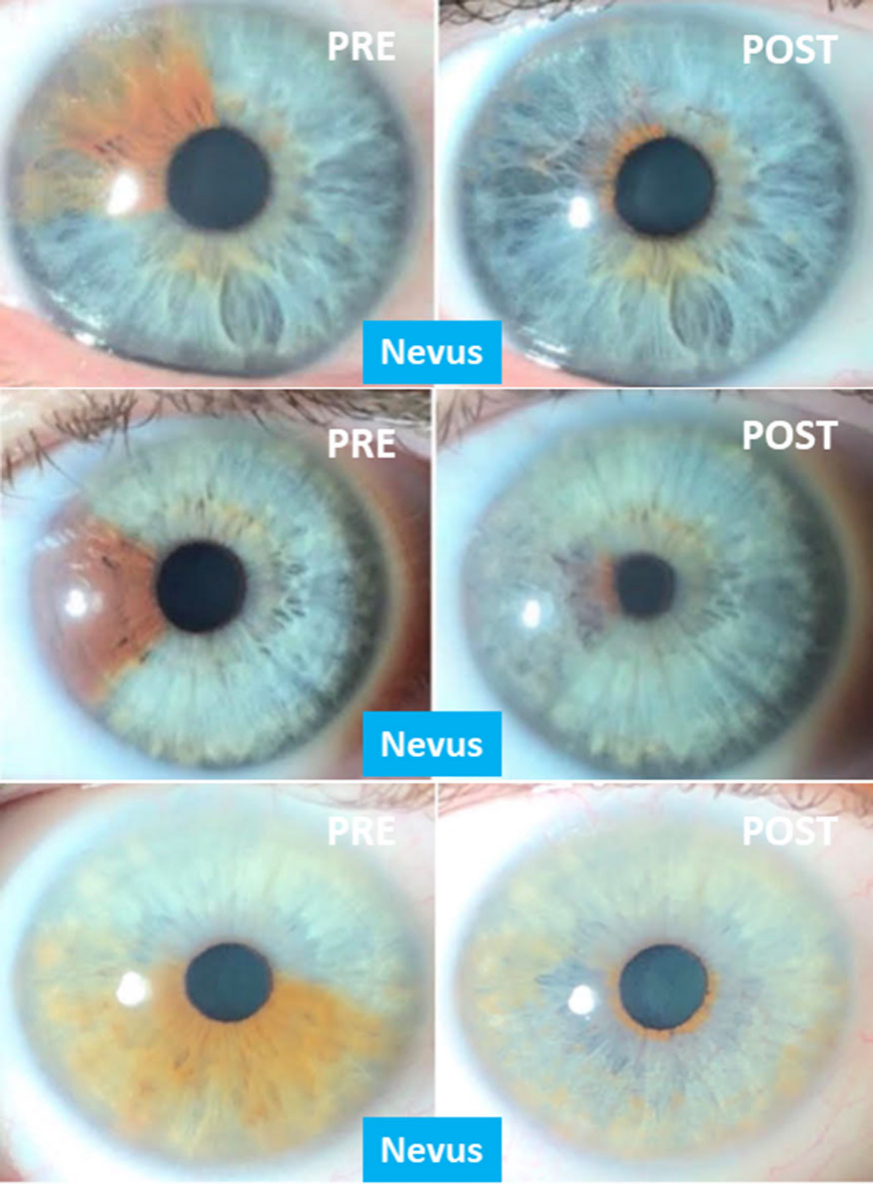
Cosmetic results in cases of nevus, before and after PCI. In this composition we can see the excellent cosmetic results obtained in three cases of large nevus, including half of the iris (below). If we achieve the removal of the pigment near the limbus and the pupil, the final appearance is almost perfect. The color underlying the original nevus is similar to the rest of the iris, so an equality is obtained between the two eyes of the patient

**Fig. 7:**
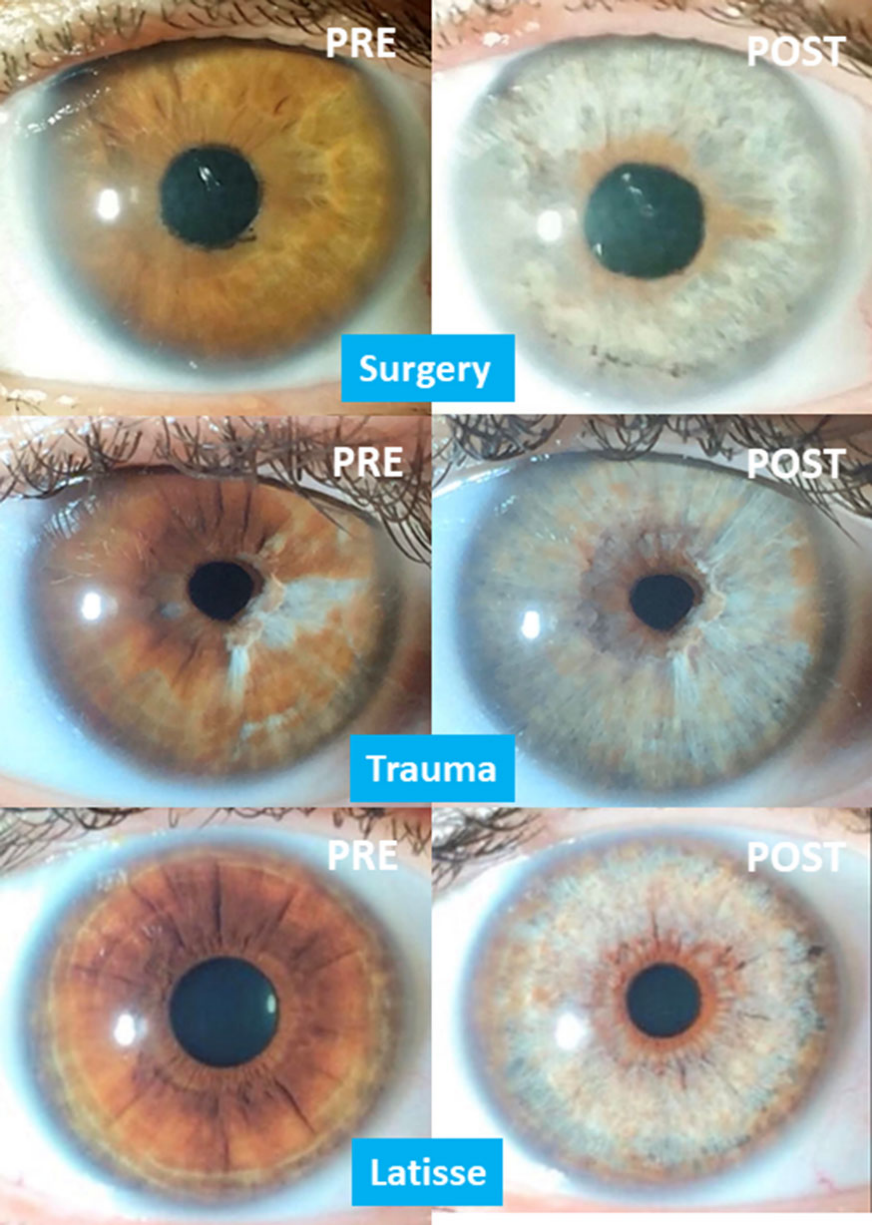
Results in complicated cataract surgery (above), cases of trauma (middle), and abuse of topical prostaglandins (down). Among the main acquired causes of heterochromia are those secondary to complicated eye surgeries, such as congenital cataracts (above), those due to eye trauma with secondary hyperpigmentation (middle), and most frequently, those secondary to the abuse of drops with prostaglandins (Latisse^®^) (below). In these indications, the PCI achieves excellent results, although after several phases of sessions, in certain patients

**Fig. 8:**
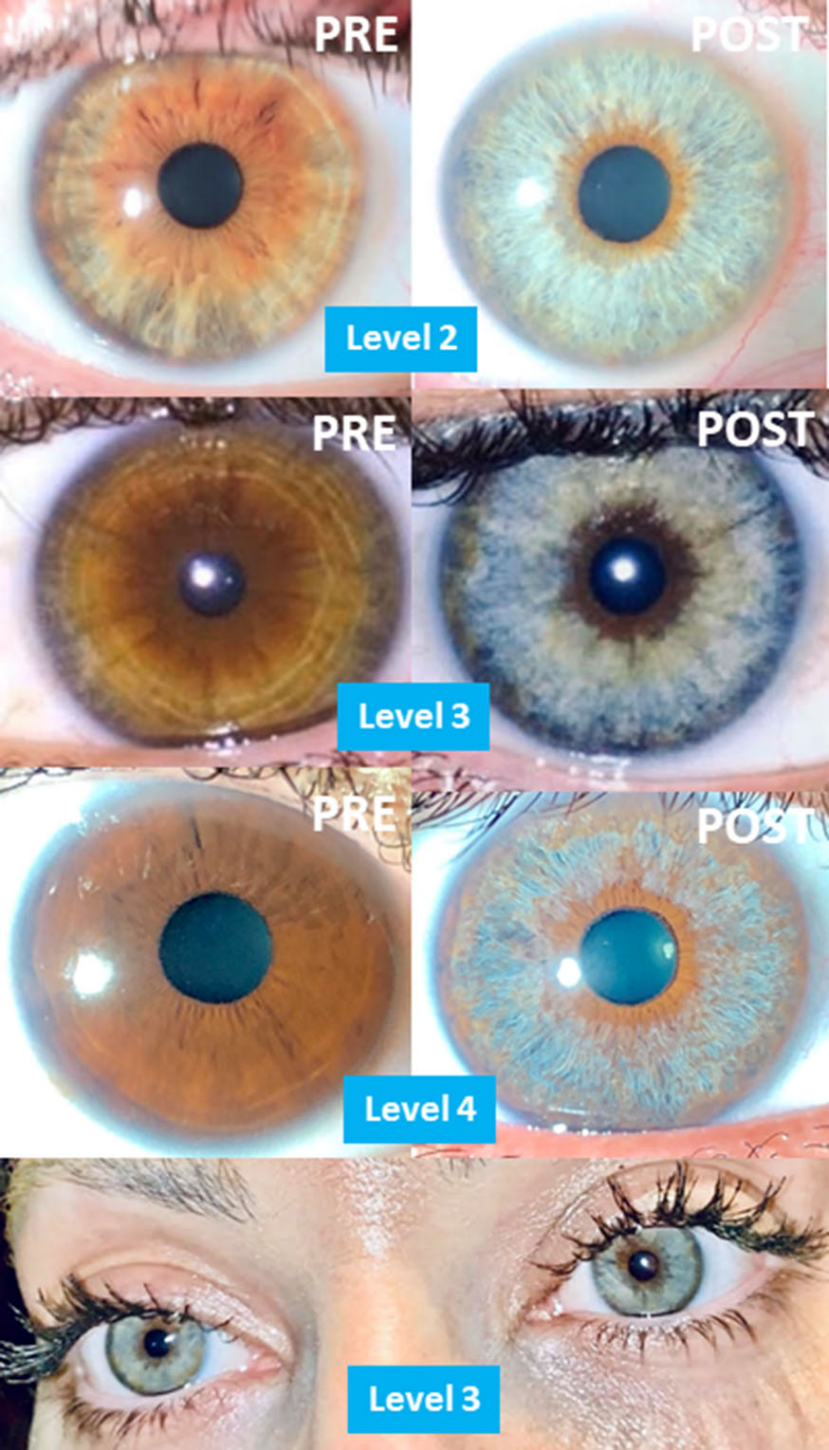
Amazing outcomes of cosmetic indications, according to degree of pigmentation: grade II (above), grade III (middle) and grade IV (down), and both eyes look from a level III look (bottom). The most frequent indication for PCI is purely cosmetic (91%). In grades I, II and III, we almost always get bluish, more or less bright results. In contrast, in highly pigmented eyes (grade IV) we usually achieve bluish-gray results. Below we see the blue look of a patient treated with grade III

Regarding aesthetic aspects, according to the indications, we obtained excellent and complete results in cases of nevus (Fig. 6), congenital heterochromia, unilateral or bilateral, partial or complete, and secondary to trauma, complex surgeries and abuse of topical prostaglandins (Fig. 7). Amazing outcomes are reached in patients with cosmetic motivations without prior pigmentary alterations (Fig. 8). For grades I, II, and III, we achieved turquoise blue in almost 100% of cases, with variations in tonality and brightness, according to the pigmentary characteristics of the patient and their parents. On the contrary, in dark eyes, grade IV, almost 100% of eyes achieved a gray blue or silver color, with varying brightness (Fig. 8). The risk of an accidental touch of the crystalline lens is minimal, with proper collaboration, so the edge of the pupil can be treated precisely. This was a highly desired requirement for the patients, along with the presence of a dark ring around the periphery, which emphasizes the contrast.

## Discussion

In our experience, photoablative cosmetic iridoplasty (PCI) is without a doubt the first-choice treatment for pigmentary alterations in the iris, given that it corrects the problem in a minimally invasive manner, at the correct level, without harming the vital structures in the stroma, collagen, elastin, blood vessels, nerves, and muscles. The 532-nm Crystal Q-switched Nd: Yag laser in 3–4 ns pulses is specifically suited to be absorbed by the melanin, as confirmed previous ultrastructural studies [23], achieving a photoablative effect due to its short discharge time, and generating microparticles thanks to its large spot size (400 µm). In no case was the posterior pigmented epithelium harmed (the UV protective layer), as verified at a histological level and using OCT.

The Crystal Q-switched Nd: Yag laser (532 nm) with 3–4 ns pulses is shown to achieve a perfect depigmentation of the anterior iris, as was suggested in other studies. However, these two previous publications refer to an experimental work in rabbits [21], and a single clinical case of heterochromia [22]. Therefore, our study is the first in the world to establish all the scientifically proven bases, in a large series of patients and with a long follow-up time of 9 years, the first 5 years to compare four different types of laser and the last 4 years performed only with 532 nm/3–4 ns Nd: Yag Laser.

Our previous research using optical coherence tomography (OCT) and pathological anatomy in cadaver eyes has shown that the elimination of the melanocytes in the superficial epithelium of the iris is selective for eumelanin (brown) and pheomelanin (yellow and ocher), due to the 532-nm wavelength, and its depth of action is very limited thanks to the short discharge duration (3–4 ns) [23]. Therefore, a photoablative effect is obtained, very similar to that of an excimer laser when the superficial layers of the corneal stroma are removed to correct myopia using PRK, or LASIK after lifting the flap. In the same way, photoablation of the pigmented layer occurs without damaging the underlying stroma of the iris, which is full of vital structures such as collagen and elastin fibers, blood vessels, nerves, fibroblasts, and muscular fibers (sphincter and dilator) [23]. The specular microscope showed no secondary effects on the corneal endothelium.

We can find numerous publications in the bibliography, starting many years ago, that have shown these unique characteristics when performing selective trabeculoplasties [18–20], emphasizing that it is harmless at a trabecular level, contrary to those performed with thermal lasers like the Nd: Yag 532 [23]. The use of thermal lasers has also been presented as an alternative, with highly repetitive micro-pulses (Micro Pulse Laser Trabeculoplasty, or MLT) [20], at 532 nm and 577 nm, but these are ineffective for iris depigmentation.

We have no doubt that PCI is much more suitable than cosmetic intraocular lenses or keratopigmentation, which have caused many very serious complications [1–13] and have also led to artificial “robot eye” aesthetic results, with fixed pupils, visual field limitations, and parasitic reflections. PCI, on the other hand, with its very high efficacy, safety, predictability, and satisfaction index, achieves cosmetic results that are always natural, with reactive pupils without any unattractive reflections.

In 2012, we had the first successfully treated case of congenital heterochromia, in a 52-year-old patient, an IT engineer, with excellent progress after 9 years. Afterward, the indications were extended to include traumatic causes, following surgery complications, iatrogenesis (due to the abuse of topical prostaglandins), simple or multiple nevus and particularly cases without prior pigmentary alterations—that is, for cosmetic reasons. It is worth mentioning that these cosmetic reasons did not tend to be merely capricious. Many patients cited professional reasons (models, actors, flight attendants), sentimental reasons, and, above all, psychological (family) or social (discrimination) reasons.

Three rules should be followed to ensure the success of PCI: perform a strict candidate selection process, a realistic prediction, and use suitable diagnostic and laser equipment for this specific treatment. This final rule is indispensable. To perform laser refractive surgery, pachymetry, topography, and aberrometry are required, as well as the excimer or femtosecond laser. Phacoemulsification requires topography, echographic or optic biometry, the precise program for calculation of intraocular lenses to be implanted, as well as the phacoemulsifier (ultrasound or laser), and the femtolaser. In the same way, the use of an specific iris scanner is mandatory for PCI, based in three modules: photographic, tomographic, and pneumotonographic, for the correct evaluation of efficacy, security and predictability, before and after the procedure. The program for obtaining a realistic prediction is also essential, as well as a software to create a 3D simulation of the achieved effect and the influence of lighting and distance. The Eyecos Iris Summary offers us a global certification guarantee that includes the efficacy, safety, and predictability of the PCI in each specific case. Finally, we will need laser equipment that meets all the requirements for the technique.

Three fundamental ideas should be clarified with the candidates. First, that added laser sessions, once treatment is complete, will not obtain a better result, but rather the opposite. Second, that the color effect depends on the ambient light, and is not very noticeable in the dark. And third, that not all patients can achieve the same results, since those depend on the initial pigmentation level, genes inherited from their patients, scarring, and many other factors like diet or exposure to sunlight.

Photoablative cosmetic iridoplasty (PCI) inaugurates a new ophthalmologic specialty, Laser Eye Cosmetics, with a large potential market. And, if we are very strict in the inclusion criteria of candidates and the appropriate technology is used, a success close to 100% is guaranteed.

## References

[1] Steinemann T, Pinninti U, Szczotka L (2003). Ocular complications associated with the use of cosmetic contact lenses from unlicensed vendors. Eye Contact Lens.

[2] Mamalis N (2012). Cosmetic iris implants. J Cataract Refract Surg.

[3] Hoguet A, Ritterband D, Koplin R (2012). Serious ocular complications of cosmetic iris implants in 14 eyes. J Cataract Refract Surg.

[4] Galvis V, Tello A, Corrales M (2016). Postoperative results of cosmetic iris implants. J Cataract Refract Surg.

[5] Shah R, Randleman J (2012). New color iris implants. Ophthalmology.

[6] Sikder S, Davis S, Holz H, Moshirfar M (2011). Complications of new color iris implantation in phakic eyes: a review. Clin Ophthalmol.

[7] George M, Tsai J, Loewen N (2011). Bilateral irreversible severe vision loss from cosmetic iris implants. Am J Ophthalmol.

[8] Arthur S, Wright M, Kramarevsky N (2009). Uveitis-glaucoma-hyphema syndrome and corneal decompensation in association with cosmetic iris implants. Am J Ophthalmol.

[9] Thiagalingam S, Tarongoy P, Hamrah P (2008). Complications of cosmetic iris implants. J Cataract Refract Surg.

[10] Garcia-Pous M, Udaondo P, Garcia Delpech S (2011). Acute endothelial failure after cosmetic iris implants (New Iris). Clin Ophthalmol.

[11] Malik S, Ghauri A, Al-Mousa R et al (2014) Removal of artificial iris implants due to bilateral angle closure glaucoma and corneal decompensation. Presented at the XXXII congress of the European Society of Cataract and Refractive Surgeons, London

[12] Mansour A, Ahmed II, Eadie B (2016). Iritis, glaucoma and corneal decompensation associated with Bright Ocular cosmetic iris implant. Br J Ophthalmol.

[13] Anderson J, Grippo T, Sbeity Z, Ritch R (2010). Serious complications of cosmetic new color iris implantation. Acta Ophthalmol.

[14] Fernández Argones L, Cárdenas Pérez FY, Piloto Díaz I, Fernández Hernández J, Padilla González CM, Obret MI (2012). Study of the effectiveness of peripheral iridoplasty with Nd YAG laser using Scheimpflug images and gonioscopy. Rev Cubana Oftalmol.

[15] Placinta IA, Martínez C, Martínez P, Molina R, Vila J (2018). Peripheral iridoplasty with argon laser and optical coherence tomography of the anterior segment in the management of acute angle closure in the 21st century. Arch Soc Esp Oftalmol.

[16] Ipsa-Callén MC, Lara-Medina J, Zarco-Tejada JM, López E, Celis J, González F (2009). Argon laser iridoplasty as treatment of the plateau iris secondary to multiple ciliary body cysts: long-term follow-up with ultrasonic biomicroscopy. Arch Soc Esp Oftalmol.

[17] Alon S (2013). Selective laser trabeculoplasty: a clinical review. J Curr Glaucoma Prac.

[18] Leahy K, White A (2015). Selective laser trabeculoplasty: current perspectives. Clin Ophthalmol.

[19] Ayele F (2018). Safety and efficacy of SLT vs ALT: short and longer term perspectives. JOJ Ophthal.

[20] Noecker R (2009) ALT, MLT, SLT. What does it matter? Glaucoma today, pp 43–44

[21] Yildirim Y, Duzgun E, Kar T (2016). Evaluation of color-changing effect and complications after Nd: YAG laser application on iris surface. Med Sci Monit.

[22] Basoglu A, Çelik U (2018). The effect of SLT laser application on iris to treat sectorial heterochromia: a promising technique. Eye Contact Lens.

[23] Soohoo J, Seibold L, Ammar D, Kahook M (2015). Ultrastructural changes in human trabecular meshwork tissue after laser trabeculoplasty. J Ophthalmol.

